# tACS of the Cerebellum and the Motor Cortex Entrains the Spiking Activity of the Cells in Motor Thalamus in a Frequency Dependent Manner

**DOI:** 10.1109/TNSRE.2025.3644746

**Published:** 2026

**Authors:** Amir Roshani Talesh, Qi Kang, Eric J. Lang, Mesut Sahin

**Affiliations:** Department of Biomedical Engineering, New Jersey Institute of Technology, Newark, NJ 07102 USA; Department of Biomedical Engineering, New Jersey Institute of Technology, Newark, NJ 07102 USA; Department of Neuroscience, NYU Grossman School of Medicine, New York City, NY 10016 USA; Department of Biomedical Engineering, New Jersey Institute of Technology, Newark, NJ 07102 USA

**Keywords:** Brain stimulation, motor thalamus, non-invasive neural stimulation, transcranial AC stimulation, trans-synaptic modulation

## Abstract

Transcranial AC stimulation (tACS) of the cerebellum can entrain spiking activity in the Purkinje cells (PCs) of the cerebellar cortex and, through their projections, the cells in the cerebellar nuclei (CN). In this paper, we investigated if the cells in the motor thalamus (Mthal) can also be modulated (i.e. spikes entrained) via the CN-Mthal projections in rodents. A total of 82 thalamic cells were found, presumably in the Mthal by their stereotaxic coordinates, that were modulated by tACS of the cerebellum. Out of the 346 cells isolated, the thalamic cells with shorter action potentials and regular firing patterns had a higher probability of modulation by cerebellar stimulation than the cells with wider action potentials. The modulation level had a tuning curve with a maximum around 100–200 Hz. Spike histograms over the stimulation cycle transitioned between unimodal and bimodal distributions depending on the frequency. Most cells had a unimodal distribution at low frequencies, a bimodal distribution for frequencies between 80–125 Hz, and then a unimodal one for frequencies above 150 Hz. In addition, tACS of the motor cortex (MC) was also tested in a subset of thalamic cells. Unlike cerebellar stimulation, modulation levels peaked at two distinct frequencies, presumably due to entrainment through multiple MC-Mthal pathways with different preferred frequencies. The results demonstrate the feasibility of modulating a deep brain structure such as the thalamus through multi-synaptic pathways by stimulation of the cerebellar cortex (and the motor cortex) using a non-invasive neuromodulation method.

## I. Introduction

TACS is a non-invasive brain stimulation technique that can entrain spiking activity of the cells by perturbing the transmembrane potential at subthreshold levels. Transcranial electric stimulation typically targets cortical circuits because the electrical field (E-field) decreases exponentially with depth [1], and this makes creating sufficient E-fields in deeper regions difficult without exposing cortical cells (as well as the cutaneous and muscle tissue) to extreme E-field levels. Transsynaptic modulation via transcranial stimulation offers a great advantage for accessing subcortical structures while keeping the E-field at the cortical level within safe levels. Transsynaptic effects of transcranial electrical stimulation are unavoidable due to extensive corticofugal projections [2], [3]. For instance, activity of the motor cortex can be modulated via optogenetic [4], [5] or epidural [6] stimulation of the cerebellar cortex. However, modulation of the cells at the post-synaptic sites is rarely considered for their therapeutic effects. In this paper, we aim to characterize the entrainment of thalamic cells through a disynaptic cerebello-thalamic pathway with AC stimulations applied to the cerebellar and motor cortices.

Motor thalamus (Mthal) in rodents, comprising the ventral anterior (VA), ventral lateral (VL), and ventral medial (VM) thalamic nuclei, receives projections from the basal ganglia, cerebellum, and reticular thalamus in addition to connecting reciprocally with motor cortex (MC) [7], [8]. The Mthal is analogous to the ventral intermediate (VIM) thalamus in humans. The VIM is the cerebellar relay nucleus that is targeted for deep brain stimulation primarily in essential tremor but also in Parkinson’s patients with residual tremor after globus pallidus internus or subthalamic nucleus stimulation [9], [10]. The connections from the PCs of the cerebellar cortex through Mthal is a major trisynaptic afferent pathway to the MC [11]. Therefore, cerebellar tACS (ctACS) can potentially offer a non-invasive alternative to deep brain stimulation (DBS) of the thalamus in the future for improving symptoms in motor disorders via modulation of the thalamo-cortical circuits projected by the cerebellar outputs.

The frequency of stimulation, as it has been tested in several human trials [12], [13], [14], [15], [16], [17], is clearly an important parameter in ctACS for efficient cerebellar modulation. Previously, we reported entrainment of PCs and transsynaptic modulation of the CN cells by AC stimulation of the cerebellar cortex [1], [18], [19], [20]. The results in anesthetized animals showed that frequencies above ~50 Hz were much more efficient for spike entrainment compared to the lower frequencies and that the PC and CN cells could be entrained at much higher frequencies than their spontaneous firing rates. In addition, the CN cell spike histograms had a bimodal distribution at certain frequencies, presumably due to two separate groups of PC projections that were excited in different phases of the sinusoidal stimulation waveform.

In this paper, we take this approach a step further by describing the disynaptic modulation of the Mthal via tACS of the cerebellum. Modulation of thalamic cells via tACS of the motor cortex (MC) stimulation is also demonstrated. The results support the feasibility and efficiency of multi-synaptic modulation of the thalamus via both cerebellar and MC tACS. Note that the word *modulation* is used synonymously throughout this paper to refer to spike entrainment, which is a special form of neural modulation.

## II. Methodology

### A. Animal Preparation.

**Fig. 1.**
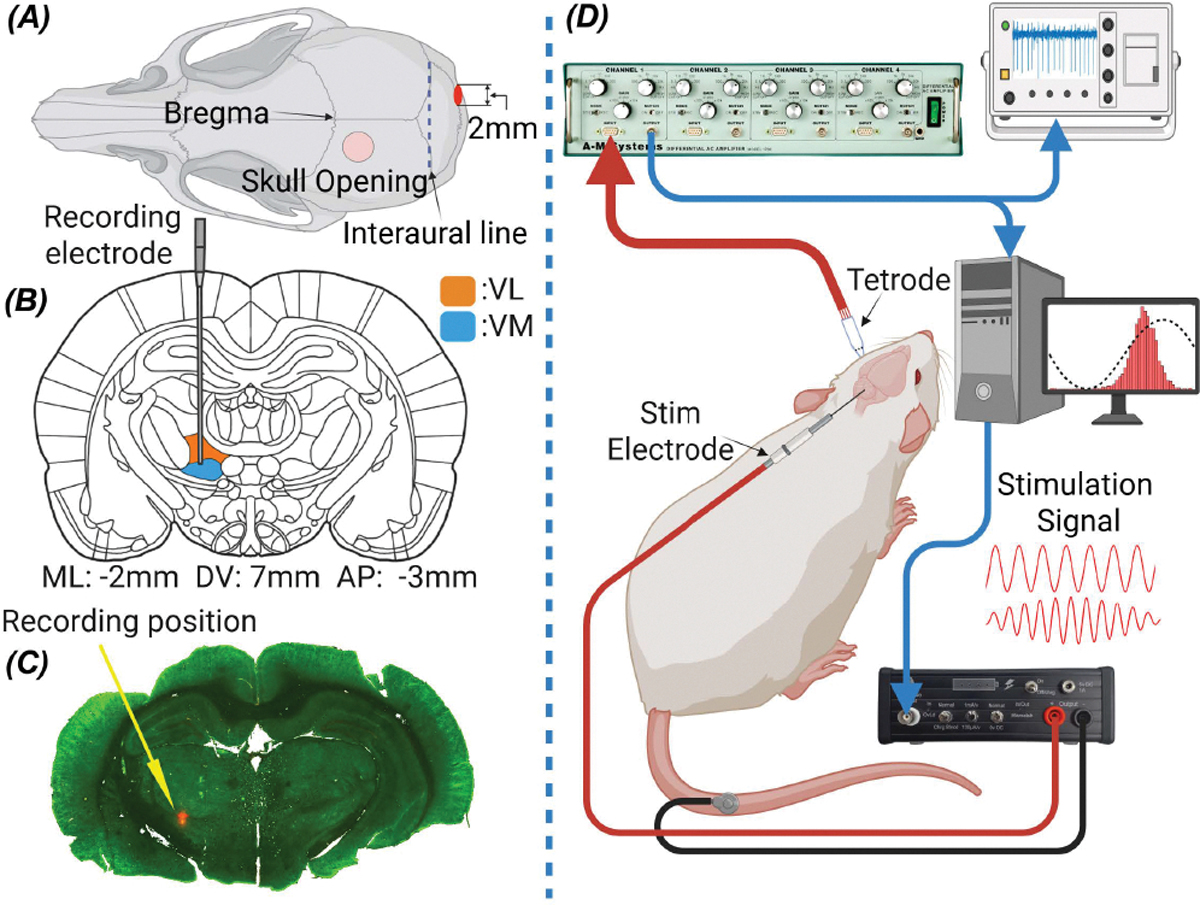
Experimental setup for recording of thalamic cells during transcranial cerebellar and motor cortex stimulation. (A) Locations of the craniotomy atop the skull for targeting the Mthal with a tetrode and the stimulation electrodes on the posterior skull and over the motor cortex are shown with small red circles. (B) VL and VM sections of Mthal are illustrated in a coronal section. (C) The tetrode tip was dyed with DiD’ leaves a mark in the Mthal in a representative case near the VL/VM border. (D) Connection of the stimulation and recording electrodes to the animal and the equipment.

Twenty-five Sprague Dawley rats (200–350g, Charles River) were used in this study. All experiments were performed in accordance with the National Institute of Health Guide for the Care and Use of Laboratory Animals. All procedures were approved by the Institutional Animal Care and Use Committee (IACUC), Rutgers University, Newark, New Jersey. Animals were initially anesthetized with 5% isoflurane in an induction chamber and then moved to a stereotaxic frame inside a Faraday cage. The animal was switched to urethane (1.35 g/kg, IP) during surgery and data collection. An additional dose of urethane (0.25 g/kg, IP) was injected as needed based on the pedal reflexes. Body temperature was measured by a rectal probe and regulated with a heating pad under the animal (ATC 1000, WPI, Sarasota, FL). The blood oxygen level, monitored with a pulse oximeter attached to the hind paw, was kept above 90% during the data collection. The hair over the head was shaved and a midline skin incision was made from bregma to the neck to expose the skull. A small cranial hole (diam: 1.5 mm) was made atop the skull for spike recording in the thalamus (Fig. 1A). In a subset of animals, the recording position was marked with 10% DiD’ solution (Cat# D7757, Invitrogen) by replacing the tetrode with a glass micropipette and pressure injecting 0.05 *μ*L of the solution at the same coordinates of the tetrode tip. The animal was then perfused transcardially with normal saline (200 ml, 60 mL/min) followed by 10% formalin (200 mL). Coronal slides of 40 *μ*m thickness were prepared for single-plex immunohistochemistry. Sections were stained using the rabbit polyclonal Anti-NeuN antibody (ABN78, Millipore Sigma) as the primary antibody and Goat anti-Rabbit IgG (Alexa Flour 488, Cat #A-11008, ThermoFisher Scientific) as secondary antibody. The confocal images confirmed the recording positions to be in the ventral thalamus (red dot in Fig. 1C).

### B. Stimulation of Cerebellar Cortex.

Stimulus waveforms were generated in MATLAB (Math-Works, MA), sent out through a data acquisition board (PCI-6255, National Inst, Austin, TX), passed through a V/I converter (Linear Current Isolator 1208HV, Caputron, Hillsborough, NJ) to generate the stimulus currents, and applied transcranially through silver electrodes (Fig. 1A).

For cerebellar stimulation, a 2 mm diameter disk electrode (Ag/AgCl, EP2, WPI, Sarasota, FL) was applied on the right side of the posterior skull over the paravermal crus II contralateral to the recording electrode. For MC stimulation, a silver wire (0.5 mm diameter) with the tip sanded with 220 grit paper to obtain a flat circular footprint, and coated with AgCl by immersing it in 1.84% sodium hypochlorite solution overnight, was applied atop the skull over the MC. The electrodes were insulated with heat shrink tube except the flat tip to prevent spreading stimulation current sideways. The tip was covered with conductive gel and pressed against the skull to make a firm contact. Any fluids accumulating around the stimulation electrode were removed using Q-tip cotton applicators. A disposable ECG electrode was attached to the tail as the return electrode for the stimulus currents.

### C. Data Collection.

**Fig. 2.**
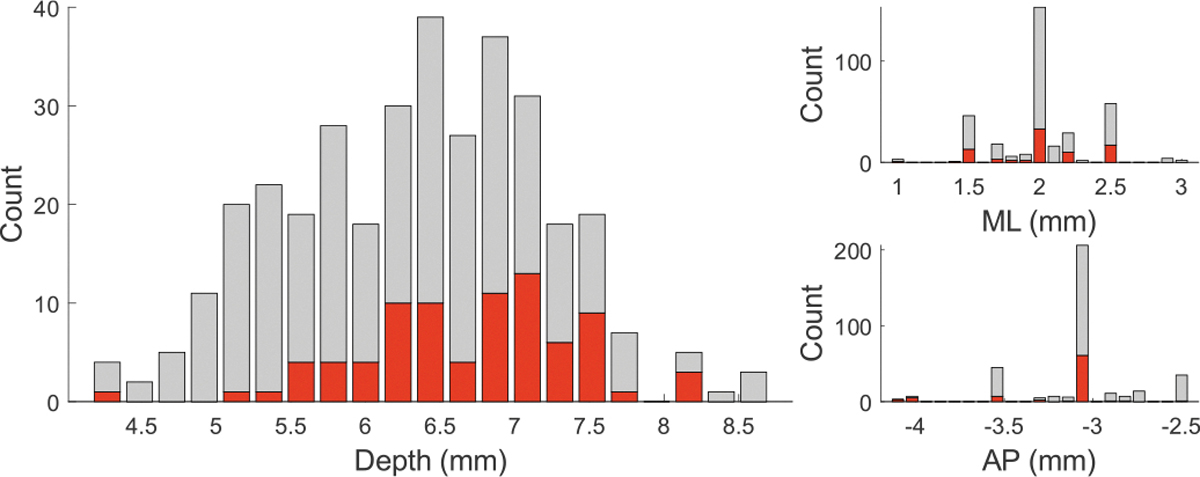
Stereotaxic coordinates (depth, mediolateral-ML and anteroposterior-AP) of 346 cells recorded in the Mthal. The cells that are modulated by cerebellar stimulation (connected) are shown in red.

Extracellular recordings in the motor thalamus were obtained using tetrodes(1–2 MΩ, AN00021, Thomas Recording Scientific Resources, Germany) in most experiments, and in some cases, glass micropipette electrodes (3–5 MΩ, filled with normal saline) were used. (Fig. 1). The electrode was inserted vertically down through a craniotomy hole. Out of 346 cells recorded in 25 rats, 82 (in 23 rats) were found to be modulated by cerebellar stimulation as determined by PLV (see below for definition) [21]. The stereotaxic coordinates of all the thalamic cells were (mean ± std): AP=−3.0±0.31 mm, ML=2.0±0.33 mm from the bregma and the depth=6.4±0.85 mm from the dura (Fig. 2). The ML and AP coordinates of the cells show a discrete (250 *μ*m increments) and non-uniform pattern because while searching for a new cell each time, the starting positions were chosen based on where the modulated cells were found in prior experiments, whereas the depth measurements had the resolution of the micromanipulator (10*μ*m).

Cerebellar AC stimulation was fully tested in 43 of the 82 modulated cells (from 19 rats) and these cells were used for subsequent AC sweep analyses. MC stimulation was tried in 15 cells (in 6 rats) out of which 5 cells were also modulated by cerebellar stimulation.

Neural signals were filtered at 100 Hz – 10 kHz, amplified(Model 1700, A-M System, Carlsborg, WA) by a gain of 100 or 1000, and sampled at 100 kHz onto the computer via data acquisition board (PCI-6255, National Instr.), while being monitored simultaneously on an oscilloscope and an audio speaker. The trials consisted of 60–120 s of stimulation at frequencies ranging from 1 Hz to 1000 Hz. For experiments involving AC sweep, the stimulus current amplitudes for the cerebellum were set to a value (200±47 *μ*A) that produced a moderate level of spike modulation. For motor cortex stimulation, the current range was 300±84 *μ*A.

### D. Data Analysis.

All data processing was performed in MATLAB. Initially, stimulation artifacts were removed by taking the stimulus-triggered average of the raw signal in a moving window with a length of 40 cycles of the stimulation to extract the artifact waveform and subtracting it from the raw signal in each cycle within the window. This method was effective even in cases where the stimulus artifact was a distorted sinusoidal with harmonics. In a few trials, a notch filter at the stimulation frequency was utilized when the moving average method did not completely remove the artifact because of sharp changes in the artifact amplitude but had no harmonics. The resulting signals were then band-pass filtered (100 Hz – 10 kHz) again on the computer.

**Fig. 3.**
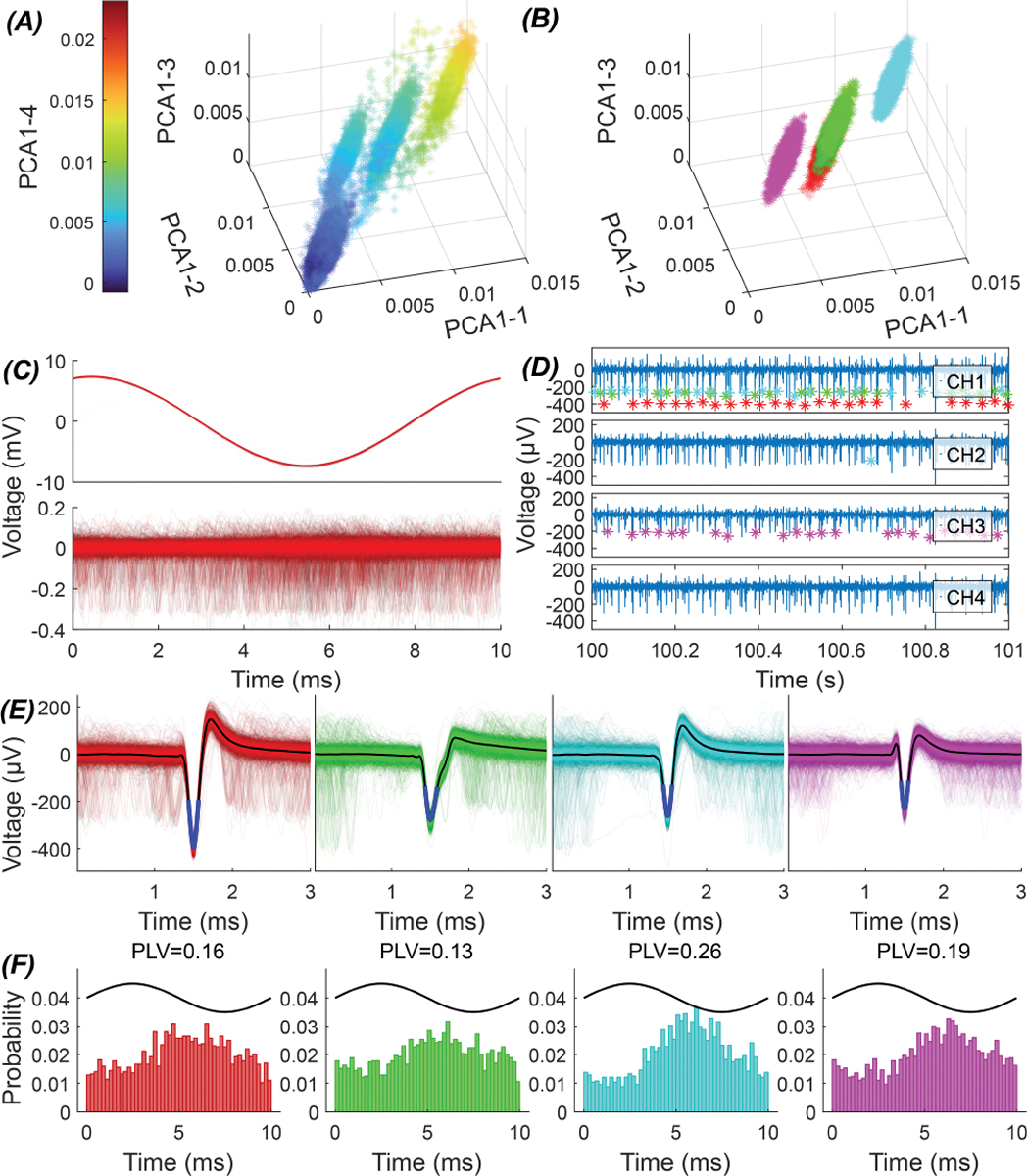
Clustering of spiking activity for a typical recording from the thalamus with a tetrode. (A) Scatter plots of the first principal component from each of the tetrode channels before clustering. PC1 of channels 1–3 are plotted along the x, y, and z axes, and PC1 of channel 4 is encoded by the color scale. Dark blue cluster in panel *A* is background noise. (B) Scatter plots after selecting 4 clusters where each cluster in different color belongs to a different cell. (C) Aggregate of raw data within the stimulation cycle (top) and after removing the extracted stimulation artifact and filtering (bottom). (D) Firing patterns of the thalamic cells in all four channels of the tetrode. Cells belonging to different clusters are marked with asterisks in different colors. (E) Aggregate of action potentials belonging to different clusters, plotted by spike triggering. (F) Probability distribution (histogram) of spike timings locked to the AC stimulation cycle for each cluster.

Spikes from the four tetrode channels were first detected by thresholding(3×estimated noise level by mediansigma=median(|noise|)/0.6745) [22], [23]. When spikes occurred within 0.5 ms in multiple channels, they were assigned to the channel with the largest amplitude to avoid double-counting the same event. For each detected spike, the action potential waveform within a ±0.75 ms window around its negative peak was extracted from all four channels and entered into principal component analysis (PCA). The first principal component from each channel was then used as an input to a Gaussian mixture model (MATLAB) to automatically select the cell clusters. With glass electrodes recordings, the first four principal components from the single channel were used as inputs to the Gaussian mixture model. From these selected clusters, well-isolated single units were identified (Fig. 3). Separation of units was verified by examining the ISI return map and the ISI histogram for each cell showing no contamination from other cells or noise.

To ensure continuity of identified units across trials, cluster parameters (centers and covariance) from the Gaussian mixture model were saved and applied in subsequent trials. In addition, continuity was confirmed by verifying that cluster positions in PCA space remained stable over the recording session, average spike waveforms did not change across trials, and ISI histograms and amplitude distributions remained consistent over time.

After separating the action potentials into clusters based on the principal components, the spike time points of the selected clusters were plotted with respect to the stimulus phase as a histogram (Fig. 3F). The mean phase vector was defined as $\text{MPV}=\text{mean}\left({\text{e}}^{\text{i}\theta }\right)$ where θ are the phase angles of individual spikes with respect to the stimulation cycle. The phase locking value (PLV) [21], [24] is the magnitude of the MPV and the preferred phase (PPh) is the phase of the MPV. The PLV was computed in two different AC cycle frames that correspond to the AC stimulation frequency (PLV1) or twice the frequency(PLV2). PLV2 was introduced because in some cases the spikes accumulated at two different phases of the AC cycle that were~180° apart (bimodal distribution) and this led to cancellation of their vectors and misleadingly low PLV1s. We categorized the distribution as unimodal if PLV1 is larger than PLV2 and otherwise as bimodal (see below for a discussion on PLV).

If a thalamic cell is modulated by cerebellar stimulation, it is modulated presumably via the axonal projections from the CN and will be referred to as a cerebellar connected cell in this study. It is important to determine the PLV threshold for classifying a thalamic cell as such. This problem can be framed as identifying the threshold PLV that it is probabilistically unlikely for a set of random spike timings to produce a greater value than this threshold. To determine the threshold, the PLV was calculated from a computer-generated set of random phases with uniform distribution, and the process was repeated a large number of times (1,000) to obtain a distribution. Then, the 99th percentile value of the distribution was taken as the threshold. This process was repeated for different number of data points (N, number of spikes), the parameter that primarily determined the PLV threshold. By fitting a curve to the threshold values for different N, we derived an analytic formula for the threshold: *PLV*_*Threshold*_ ≈2.1×N^−0.5^ (Suppl. Fig. 1A).

To validate these simulations empirically, we also found the thresholds from the spike data from all the cells recorded in this study. For most of these cells, recordings without AC stimulation were not available. Instead, the periodicity induced by AC stimulation in the spike timings was disrupted in the 100 Hz stimulation data by using prime numbers to segment the spike trains into hypothetical AC cycle lengths that are not exact multiples of 100 Hz cycle (10 ms), and PLVs were computed for different cycle lengths. We then computed the 99^th^ percentile value for the PLVs distributed randomly from all the cells for different data lengths (N) and fitted a curve to this empirical data as we did in simulations (Suppl. Fig. 1B). The empirical curve closely matched the one derived from simulations, confirming that the threshold is reproducible across different firing patterns and spike distributions. The only parameter that made a difference in PLV plot was the number of spikes, and not even the simulated AC cycle length.

For inclusion of the cells into the connected group, we compared either PLV1 or PLV2, whichever is greater, against 2 × *PLV*_*Threshold*_, as a conservative value. However, using a lower threshold (*PLV*_*Threshold*_) did not alter any of the main conclusions of this study on cellular classification. Interestingly, the classification results were so robust that even applying a constant threshold (PLV> 0.15, an arbitrary value that corresponds to a clearly appreciable level of modulation in the histogram) had a very small effect.

Coefficient of Variation (CV) of inter spike intervals (ISI), defined as σ(ISI)/μ(ISI) was used as a global measure of ISI variability, where σ and μ are the standard deviation and the mean, respectively. The CV2, defined as $CV2_{i}=2\left|ISI_{i+1}-ISI_{i}\right|/\left(ISI_{i+1}+ISI_{i}\right)$ averaged over the entire trial, was used as a measure of spike-to-spike variability of ISI, the larger value of which indicates irregularity in spiking activity. The ISI histogram peak location varied substantially from cell to cell, and thus the mode of ISI was used to differentiate between the cellular firing patterns in addition to CV and CV2. Since the ISIs distributed in a large range, they were plotted using the logarithmic scale:LMI=log(Mode(ISI)). In our recordings, a very low LMI value was indicative of a bursty firing pattern where the ISI histogram had a peak at small ISI values corresponding to clusters of a few rapid spikes separated by long intervals in time. We refer to this pattern as an irregular firing. Conversely, cells with higher LMI values exhibited more tonic and steady firing, referred to as regular firing pattern.

## III. Results

### A. Thalamic Cell Entrainment by ctACS.

**Fig. 4.**
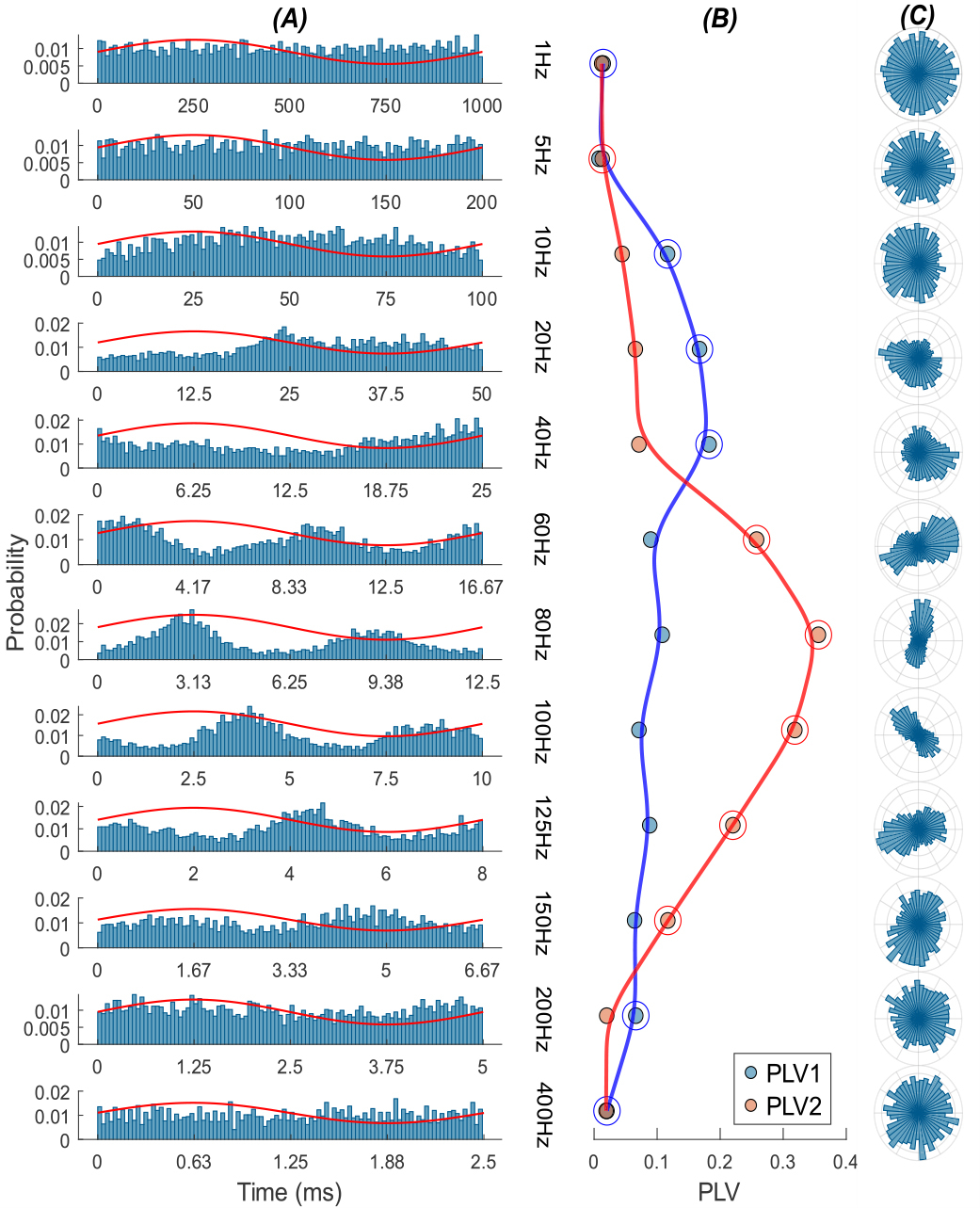
Response of a sample thalamic cell to AC sinusoidal stimulation of the cerebellar cortex with frequencies ranging from 1 Hz to 400 Hz at 200 *μ*A. (A) Probability distribution of spikes over the AC stimulation cycle at each frequency. Red traces are the stimulus current waveforms (arbitrary units). (B) Corresponding PLV1 and PLV2 at each frequency.(C) Probability distribution of spiking activity in the polar plane.

Fig. 4 shows a thalamic cell that responded to transcranial AC stimulation of the cerebellum. The histograms show a bimodal response(PLV2 >PLV1) at mid-frequencies and a unimodal response (PLV1 >PLV2)at low and high frequencies.

**Fig. 5.**
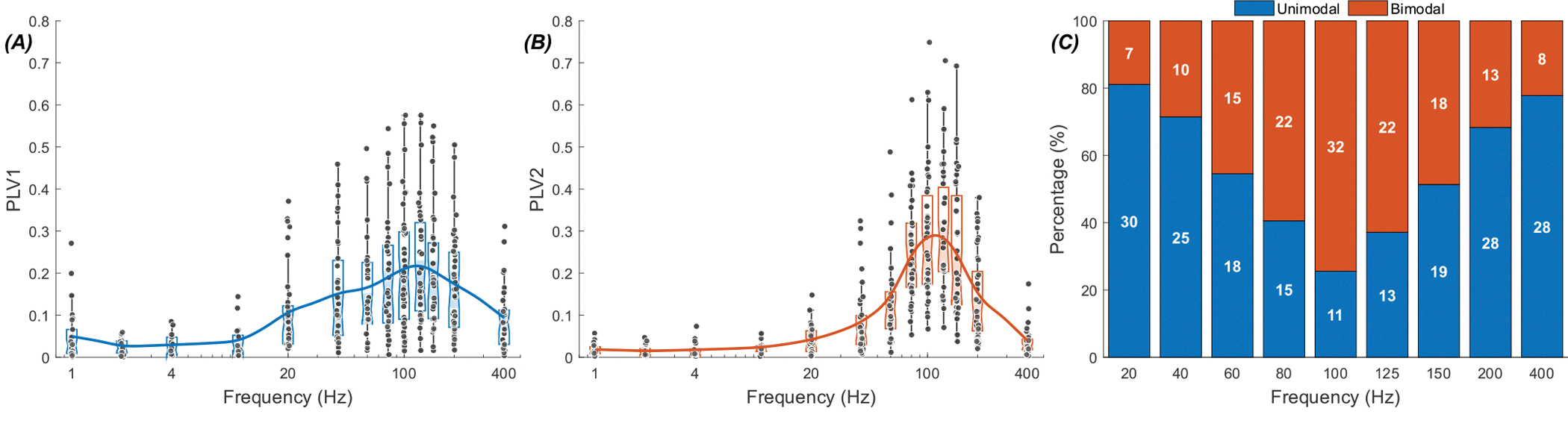
PLV-frequency plots for thalamic cells during stimulation of the cerebellum (43 cells in 19 rats). (A)PLV1. (B) PLV2. (C) Percentage of unimodal responses(blue) and bimodal responses (red) at each frequency, with the number of cells indicated. For the unimodal case, Wilcoxon signed-rank comparisons between the peak frequency (125 Hz) and the other tested frequencies showed significant differences after Holm–Bonferroni correction. PLV at 125 Hz exceeded values at 1–60 Hz and 400 Hz (all adjusted p <0.04), with consistently large effect sizes (r >0.59), highlighting a significant peak at 125 Hz. In the bimodal case, significant differences were again observed, with PLV at 125 Hz greater than at 1–60 Hz and 150–400 Hz(all adjusted p < 0.008). The correlation coefficients were large (r > 0.74), further confirming a robust peak response at 125 Hz. (The effect size in Wilcoxon signed-rank is based on correlation coefficients, here and inFig. 11 [25]).

Fig. 5 presents the PLV measurements as a function of frequency in a group of 43 cells from 19 rats (panels A & B) and shows the percentage of cells with bimodal and unimodal responses at each frequency (panel C). The group averages peak around 125 Hz in both PLV1 and PLV2 plots. As in Fig. 4, PLV2 is larger in mid-frequencies than PLV1, suggesting that the modulation is predominantly a bimodal one. The dominance shifts to unimodal distribution at the lower and higher ends of the spectrum (PLV1 >PLV2). The maximum number of bimodal responses occur at 100 Hz (32 vs. 11, right panel), about where the PLVs also peak.

PPh was computed to see if the PC-Mthal propagation delay could account for the phase shift in modulation with increasing frequency. The PPh based on the whole cycle was used for the histograms that are classified as unimodal (PLV1 >PLV2). For bimodal cases, the PPh was calculated for each histogram peak separately (See Suppl. Fig. 2 for examples of these cases).

**Fig. 6.**
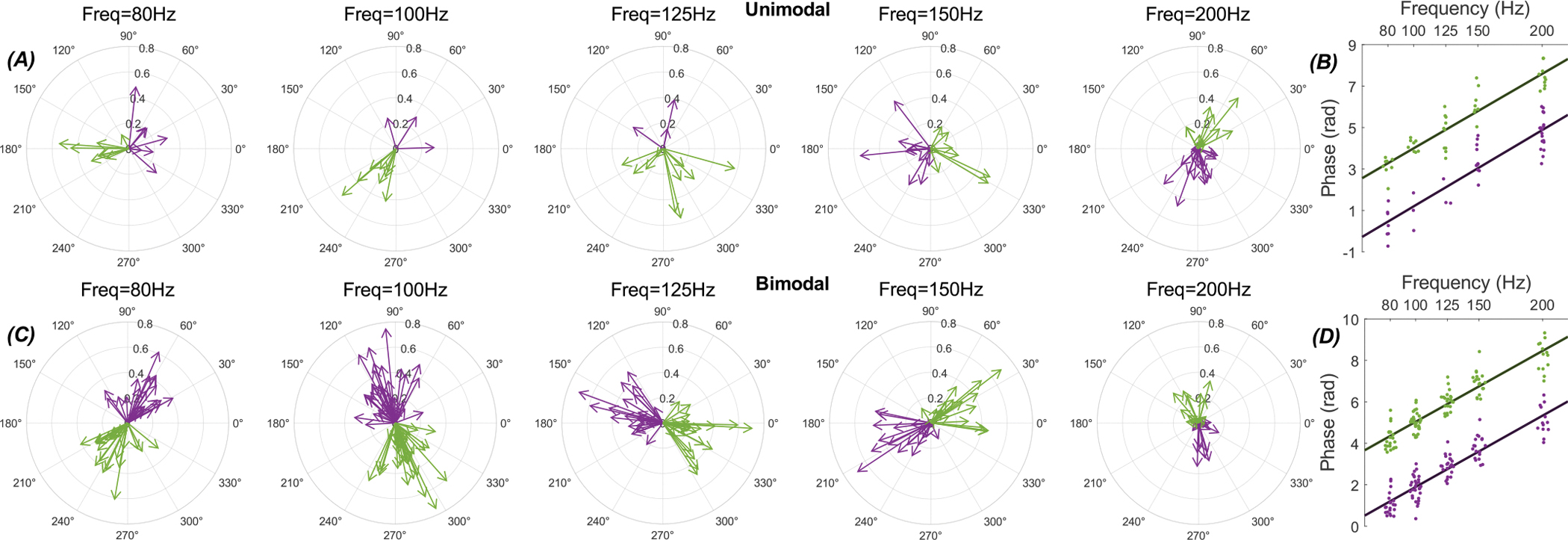
Mean phase vectors of the response to AC stimulation (80, 100, 125, 150 and 200 Hz) in thalamic cells (43 cells in 19 rats). (A-B) Unimodal. (C-D) Bimodal. B and D show the average phase shifts for each vector group, coded in different colors, as a function of frequency. A linear line-fit to the phase shifts has a slope of 0.034 rad/Hz for the bimodal peaks (D) and 0.036 rad/Hz for the means of the vector groups of the unimodal responses (B). The different colors indicate two groups of vectors classified into separate clusters by k-means clustering. Unimodal cases were represented with a single vector, while the bimodal cases were represented with two vectors, one for each peak in the histogram. To cluster the vectors into two groups, k-means clustering was repeated 1000 times, and the most frequently occurring centers were taken as the final cluster centers. In the bimodal case, we ensured that the two vectors of the same response were not placed in the same cluster. The only assumption made was that the phase rotates counterclockwise with increasing frequency, consistent with the expected propagation delay, and the cluster centers were assigned based on this assumption. For the unimodal cases, the number of vectors is the number of unimodal responses at the given frequency, as marked in Fig. 5C, whereas for bimodal cases, the count is doubled since each histogram was represented with two vectors.

Fig. 6 shows the MPVs for unimodal and bimodal histograms of all the cells included in Fig. 5, at mid-frequencies (80 Hz- 200 Hz), where the PLV is higher than elsewhere and thus expected to produce more accurate phase estimations. The individual peaks in the bimodal responses presented a linear change in phase as a function of frequency (Fig. 6D) that can be accounted by the propagation delay. The slope of the linear line-fit to the phase values from either one of the histogram peaks in the bimodal response was~0.034 rad/Hz. Then, the cerebello-thalamic delay was estimated to be around 5.4 ms by dividing the slope of the linear line-fit by 2*π*.

The unimodal phases at first sight presented a large degree of randomness(top row) with no apparent monotonic increase by frequency. However, when we split the vectors from individual cells into two groups with opposite phases, as we have done with the bimodal responses with double peaks, the phase was again changing linearly as a function of frequency in each group with a similar slope (~0.036 rad/Hz, Fig. 6B). This suggested that the thalamic cell responses at the population level are primarily bimodal even if the individual cells may apparently have a single peak in their histograms.

### B. Classification of Thalamic Cells.

**Fig. 7.**
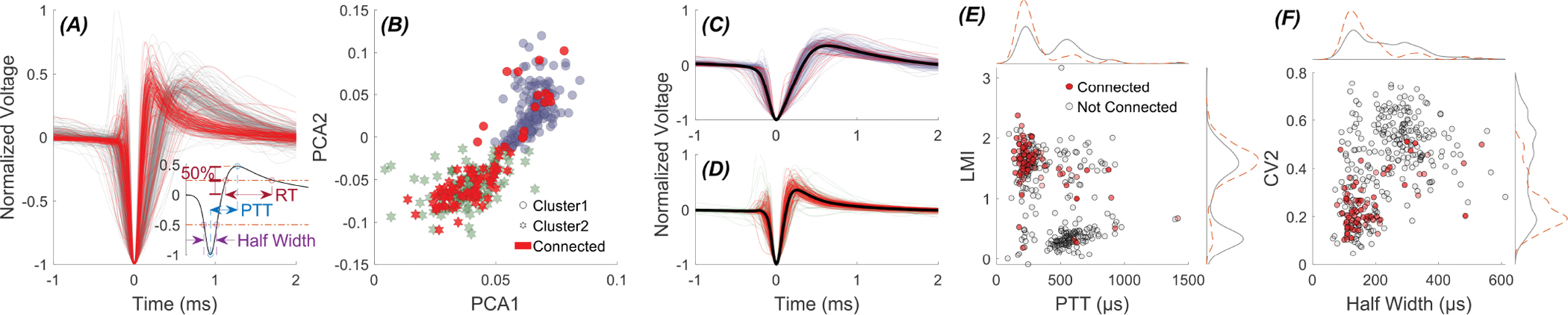
Clustering of thalamic cells (346 cells in 24 rats) based on spike-timing and -shape based parameters. (A) Action potential waveform of 346 cells (modulated cells are plotted in red). Inset: Definitions for Peak-to-Trough Time (PTT), Repolarization Time (RT) time, and Half-Width. (B) k-means clustering by the first and second principal components can divide the cells into two clusters (circles and hexagrams). (C) Action potential shapes for the first cluster. (D) Action potential shapes for the second cluster. (E) distribution of cells according to LMI and PTT. (F) distribution of cells according to CV2 and half-width. The cells that can be modulated by cerebellar stimulation marked in red in all plots.

Out of a total of 346 thalamic cells recorded, 82 cells were identified as being connected (PLV> 2 × *PLV*_*Threshold*_) to the cerebellum, as described in the Methodology section. Fig. 7A displays the action potential waveforms from all recorded cells, with connected cells shown in red and others in gray. It is evident that there are two distinct groups of action potentials based on their shape. PCA of these waveforms supports this distinction, revealing two apparent clusters in PCA space (Fig. 7B). The k-means clustering can identify two clusters where one cluster (circles, 170 cells)contains only 16 connected cells (~20% of all connected cells), while the other cluster (hexagrams, 176 cells) contains 66 connected cells (~80%). Thus, the cluster with narrower spikes (Fig. 7D) has a four times higher chance of representing the thalamic cells that are modulated by cerebellar stimulation. The Fisher’s Exact Test rejects the null hypothesis of equal membership for the connected cells, suggesting that the probability of finding connected cells is significantly higher(p= 3.2 × 10^−10^, odds ratio = 5.78, 95%CI [3.2,10.5]) within the cluster of cells with narrower action potential than the cells with wider action potentials.

Interestingly, this clustering pattern extends beyond the spike shape to parameters defined by temporal patterns of spikes. For instance, a similar pattern emerges when clustering of cells based on parameters related to spike timing, such as LMI and CV2, is compared to clustering based on shape parameters, like Peak-to-Trough Time (PTT) and spike half-width (see inset in Fig. 7A for definitions). Fig. 7E illustrates the distribution of the cells according to LMI and PTT. As expected, PTT divides the cells into two clusters (two peaks in histogram above the figure) with the cluster of smaller PTTs having a larger population of cells being connected to the cerebellum. LMI also divides the cells into two clusters where the cells with higher LMI have a greater likelihood of being connected to the cerebellum than the cells with irregular firing patterns.

A similar pattern is observed when the cells are mapped by CV2 vs. half-width, another pair of parameters derived from spike timings and spike shapes, respectively (Fig. 7F). The resulting clusters closely resemble those obtained through PCA-based clustering. For instance, k-means clustering using CV2 and half-width yields a cluster composition in which ~90% of the cells overlap with the clusters made by PCA. In comparison, the overlap of clustering using LMI and PTT is around 97%.

The characteristics of the cells in two clusters are sufficiently different that we can separate the cells only by one variable. Suppl. 3 shows the distribution of different parameters derived from the shape or firing pattern of action potentials. For instance, LMI or PTT (or RT) seem to have the ability to segregate them into distinct clusters.

These findings strongly suggest the existence of two distinct types of thalamic cells: one with broader action potential and a more irregular firing pattern, and another with narrower action potential and a regular firing pattern, where the second cluster has a significantly higher percentage of cells modulated by cerebellar stimulation.

**Fig. 8.**
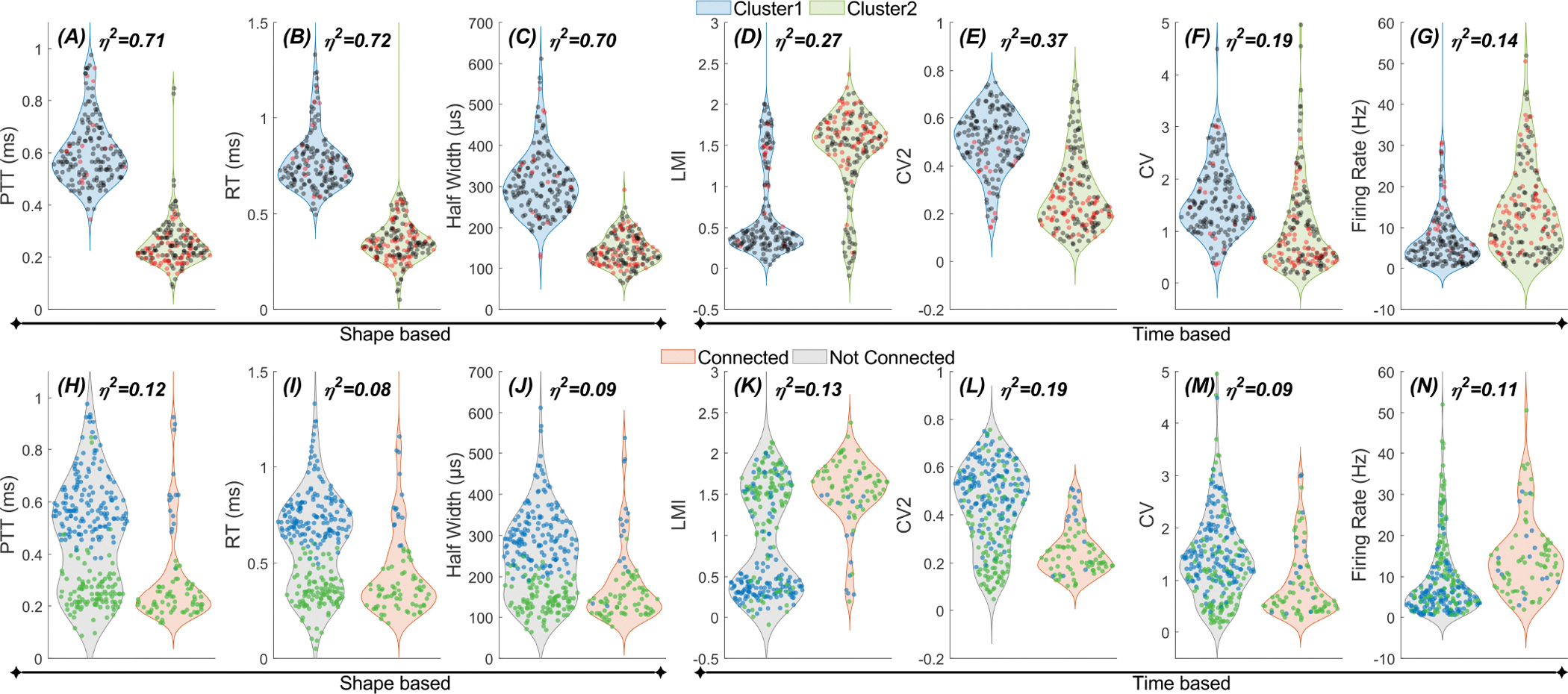
Distribution of shape and timing parameters within clusters created by PCA (top row, cluster 1 and 2) and groups by connectedness to the cerebellum (bottom row). *(A-G)* Distribution of cells within Cluster 1 and Cluster 2 according to shape *(A-C)* and timing parameters *(D-G)*. In *(A-G)* the connected cells are marked with red dots as opposed to not-connected cells in grey. *(H-N)* Distribution of cells within the groups of connected and not-connected cells according to shape *(H-J)* and timing parameters *(K-N)*. In *(H-N)* the cells in Cluster 1 are marked with blue dots as opposed to the cells in Cluster 2 in green. The effect size (*η*^2^), the square of the correlation coefficient from Wilcoxon rank sum test, is marked in each subplot [25].

We also investigated the correspondence between the clusters produced by PCA and the shape- and timing-based parameters (Fig. 8). The top row shows that PCA separates the cells into two clusters (Cluster 1 and Cluster 2), which, as confirmed by the Wilcoxon rank sum, differ significantly in all the selected shape-based (A–C) and timing-based parameters (D–G) (p< 8.0 × 10^−12^). The agreement between PCA and shape-based parameters (PTT, RT, and half-width) is expected because PCA also uses the action potential waveform. The extension of this clustering to the timing parameters (LMI, CV2, CV, and firing rate) suggests that the cells that have different action potential shapes also differ in their firing pattern.

We also conducted a similar analysis to see if connectivity to the cerebellum could separate the cells into groups that also differ by the shape and timing parameters (lower panel in Fig. 8). All parameters were significantly different between the cells connected and not connected to the cerebellum (p< 2.0 × 10^−7^, Wilcoxon rank sum). Nevertheless, overlap between the two groups was still visible for most of the parameters. In fact, it is evident that the clusters made by PCA (blue vs. green dots) were separated within each group made by the connectedness to the cerebellum in most panels from H through N. Thus, the results in Fig. 8 agree with the earlier conclusion that there are two groups of thalamic cells in our data set that are well separated by their action potential shape and firing pattern. The cluster with short spike durations and regular firing pattern has a much higher percentage of the cells that are modulated by cerebellar stimulation than the other cluster. The ability to be modulated by cerebellar stimulation, on the other hand, does not segregate the cells (to the same degree) into groups that also differ by the shape and timing-based parameters. That is, clustering based on spike shape and firing pattern predicts a cell’s likelihood of being modulated more reliably than modulation-based classification predicts spike shape or firing pattern.

**Fig. 9.**
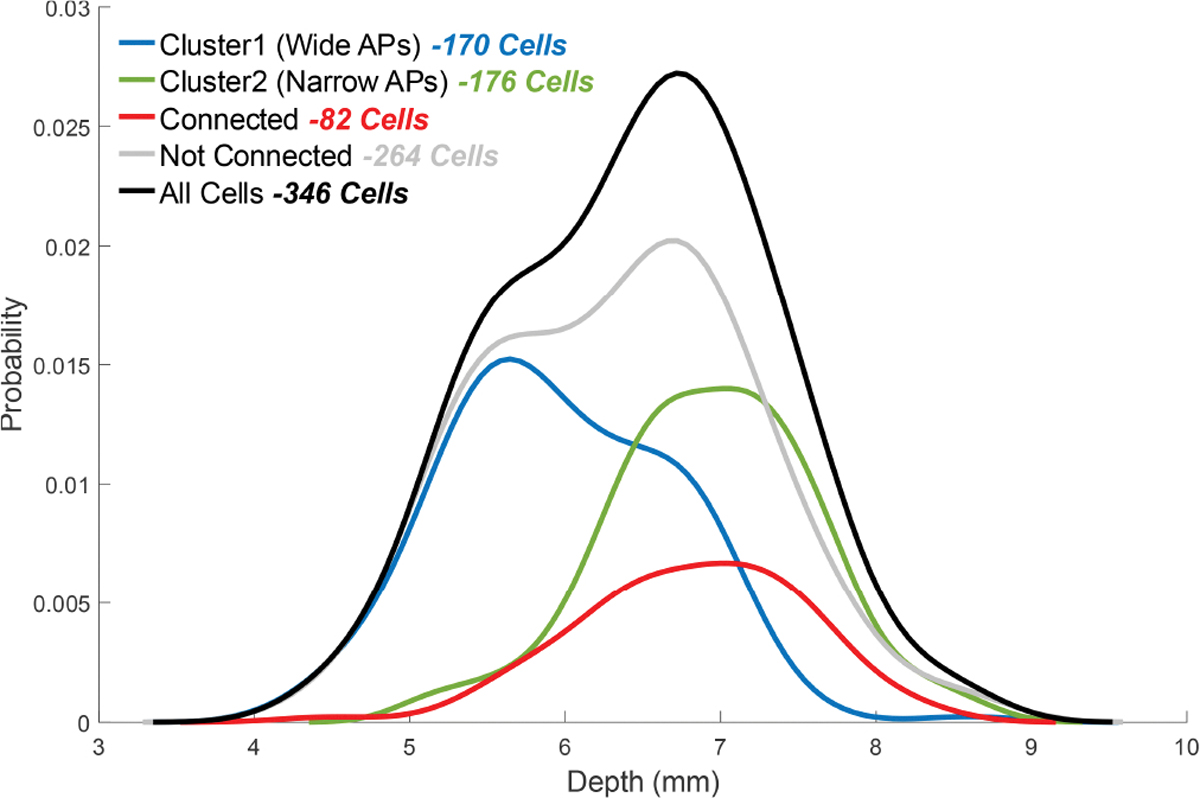
Probability distribution of thalamic cells by depth for different groups segregated according to their action potential shape (narrow vs. wide) and ability to be modulated by cerebellar stimulation (connected vs. not connected). Plots are smoothed in MATLAB using *ksdensity* with 100 bins.

The number of thalamic cells belonging to different groups were plotted according to the stereotaxic depth at which they are found (Fig. 9). The thalamic cells exhibiting narrow action potentials and regular firing patterns (Cluster 2) are typically located deeper(depth=6.9±0.68), whereas cells with broader spikes and irregular firing (Cluster 1) are more frequently found at shallower depths (depth=6.0±0.73). The Wilcoxon rank sum test confirmed a significant difference in depth between these two clusters (p = 1 × 10^−26^, z=10.7, *η*^2^ = 0.33). This depth-dependent distribution suggests that these groups represent functionally and anatomically distinct thalamic populations. Furthermore, the deeper population appears to have a higher probability of being modulated by cerebellar stimulation (connected). The Fisher’s Exact Test had showed that the odds of being connected were significantly higher for cells in Cluster 2 than in Cluster 1. Consistent with this, the depth distribution of connected cells (6.8 ± 0.71 mm) is closer to that of the Cluster 2 cells than the Cluster 1 cells. These findings indicate that connected cells are closer to Cluster 2 than Cluster 1 both in terms of the shape/timing properties and the anatomical depth profiles.

### C. Thalamic Cell Entrainment by Motor Cortex Stimulation

**Fig. 10.**
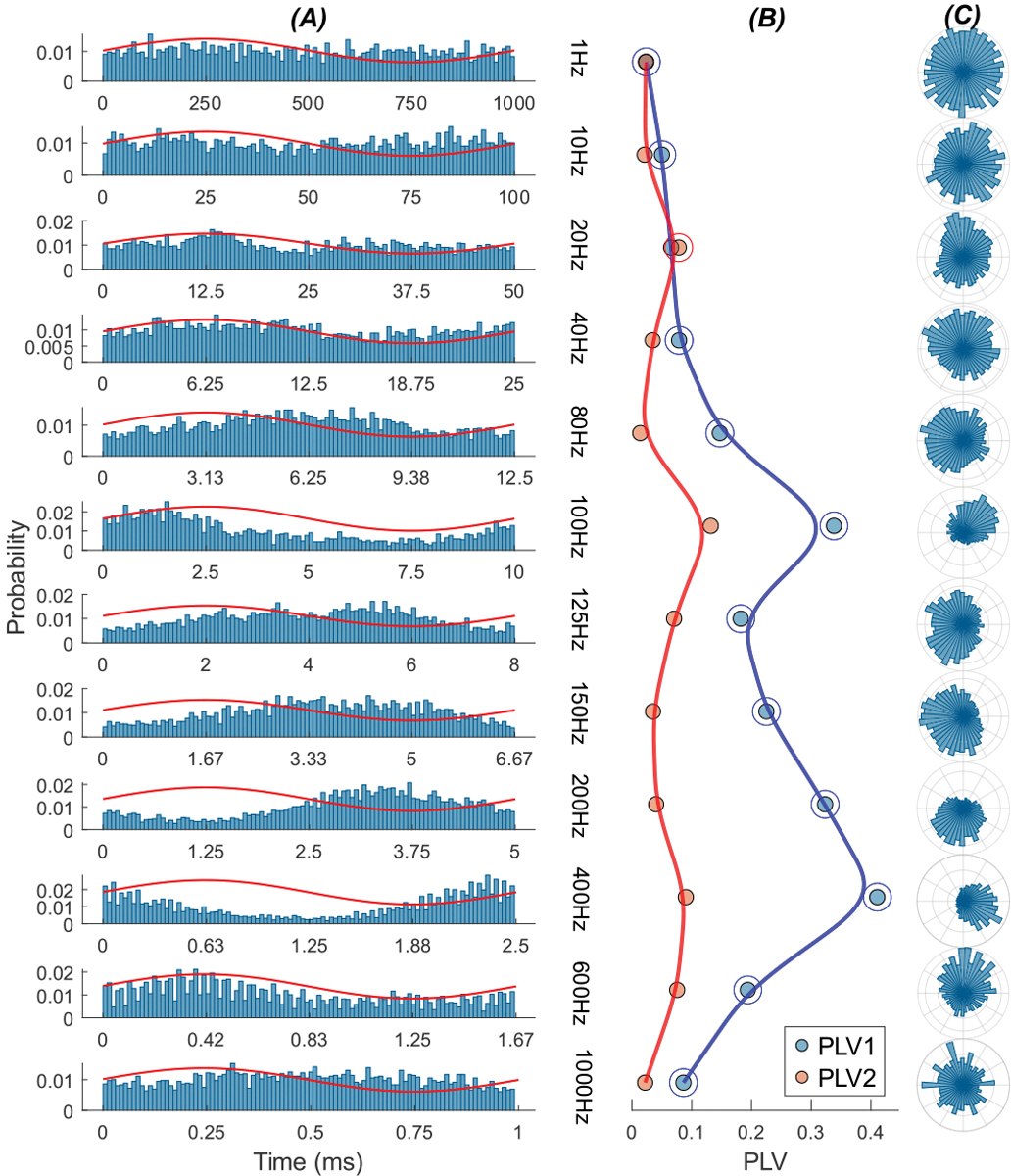
Response of a thalamic cell to sinusoidal stimulation of motor cortex with frequencies ranging from 1 Hz to 1000 Hz at 400 *μ*A. (A) Probability distribution of spikes by the AC stimulation cycle. Red traces are the stimulus current waveform in arbitrary units. (B) Corresponding PLV1 and PLV2 at each frequency. (C) Probability distribution of spiking activity in the polar plane.

Next, we investigated the possibility of entraining the thalamic cells by MC stimulation. Fig. 10 shows a typical response of a thalamic cell to MC stimulation at frequencies ranging from 1 Hz to 1,000 Hz. PLV-frequency plot differs from that of the cerebellar stimulation in that it has two local peaks. Also, the entrainment is stronger at high frequencies with a peak around 400 Hz.

**Fig. 11.**
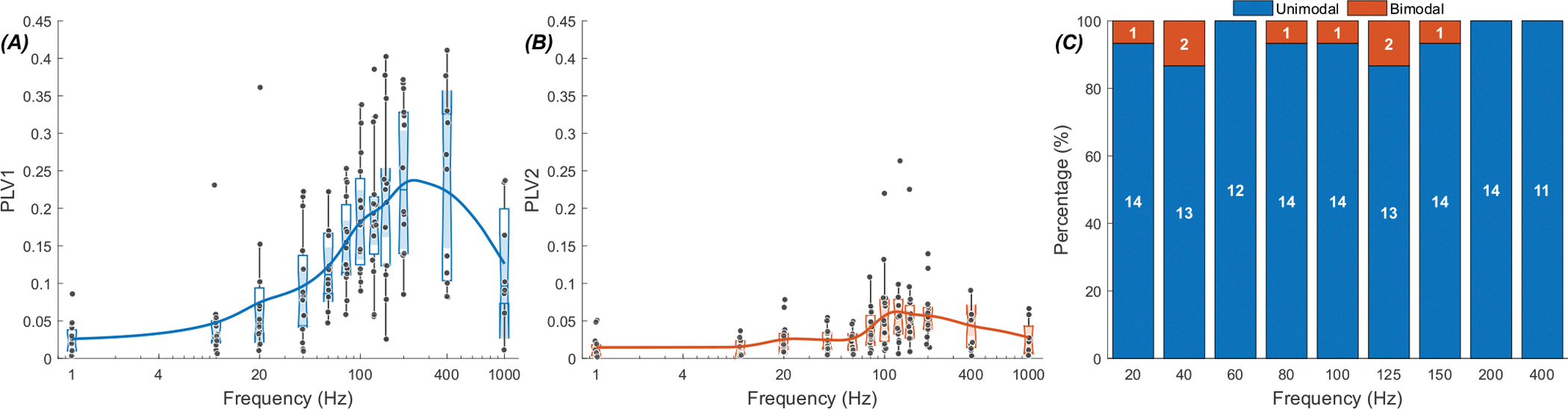
PLV-frequency plots for thalamic cells while stimulating the motor cortex (15 cells in 6 rats). (A) PLV1. (B) PLV2. (C) Percentage of unimodal (blue) and bimodal responses (red) at each frequency, with the number of cells indicated within the bars. In the unimodal case, Wilcoxon signed-rank tests revealed that the peak frequency (200 Hz) produced significantly higher PLV values than 1–80 Hz after Holm–Bonferroni correction (all adjusted p < 0.005). The correlation coefficients were r > 0.81. In the bimodal case, peak frequency (125 Hz) emerged as significantly higher than 1–60 Hz (r > 0.68, all adjusted p < 0.05).

In the group data, 9 cells out of 15 (in 6 rats) had two peaks in their PLV plots (not shown). The modulation was much stronger than cerebellar stimulation at high frequencies with a peak around 200 Hz-400 Hz (Fig. 11). Interestingly, there were very few (13 out of 164 trials at different frequencies) bimodal histograms with MC stimulation, and thus average PLV1 was larger than the PLV2 at all frequencies.

## IV. Discussion

In this paper, we investigated entrainment of Mthal cells through transcranial AC stimulation of the cerebellum and the MC. Interestingly, the Mthal cells modulated by cerebellar stimulation seem to belong to a distinct group of cells segregated by their location in the thalamus and the electrophysiological properties. The results demonstrate frequency dependent entrainment of the cells with different profiles depending on the site of stimulation. The potential mechanisms for this frequency dependency of modulation and the source of unimodal and bimodal responses are discussed in this section with supporting evidence from computer simulations. It is important to note that all results were obtained from anesthetized preparations, which limits direct extrapolation to awake or human conditions. Future studies in behaving animals are required to establish functional and behavioral relevance. Additionally, the relatively small number of thalamic cells recorded during motor cortex stimulation constrains the strength of our conclusions and should be regarded as preliminary evidence.

### A. Modulation Vs. Frequency.

A consistent observation at all sites in the cerebello-thalamic path; the Mthal cells (this paper), the CN cells [18], [20], and the PCs of the cerebellar cortex [1], has been the sharp decline of modulation levels toward the low frequencies. Anesthesia is unlikely to be the underlying cause, as two distinct anesthetic regimens—ketamine/xylazine in our previous study and urethane in the current work—were employed. Unfortunately, reports on direct cerebellar surface stimulation [19], [26] have not tested ctACS in a large range of frequencies, as we have done in this paper, to observe the full spectrum. The tuning curve observed at the PC level —characterized by reduced modulation at both the low and high ends of the frequency spectrum—appears to account for much of the tuning curve that we observed in the CN cells before [18], [27] as well as in the Mthal cells of this study. It was predicted by Knight that the average firing rate of an ensemble of spontaneously active neurons with random phases is proportional to the time derivative of the membrane potential [28]. A large dV/dt simply implies that there will be a higher number of cells reaching the threshold of firing, hence transiently a higher firing rate for the population. This concept can also apply to a single neuron when the spiking data with a randomly distributed ISI is collected over many cycles of AC stimulation, analogous to the data from an ensemble of neurons. The dV/dt of the transmembrane voltage increases with increasing frequency of the sinusoidal stimulation (if we assume that the transmembrane voltage follows the extracellular voltage) and, consequently, the modulation also rises with increasing frequency.

The increase in modulation of the PC firing rates with frequency and peaking at ~200 Hz (resonance) was predicted based on a two-compartmental cell model and verified in an *in vitro* patch- clamp experiment with current stimulation to the soma [29]. The underlying mechanism for this resonance was convincingly shown to be the dV/dt phenomenon, discussed above, in addition to a flat input impedance profile up to high frequencies provided by a second compartment added to the model to simulate the dendrites. The transmembrane capacitance in series with the transmembrane resistance of the dendritic tree (which is many times smaller than the somatic resistance in PCs [30]) forms a low-pass filter that eventually starts lowering the transmembrane voltage (and the modulation) at higher frequencies.

### B. Bimodal Vs. Unimodal Thalamic Responses.

In [1], we demonstrated that some PCs are excited by the rising edge of the stimulation pulse and inhibited by the falling edge (upward PC), while others exhibit the opposite response (downward PC). Under sinusoidal AC stimulation, this phenomenon causes the responses of these two PC groups to be phase-shifted by 180° relative to each other. The unimodal and bimodal responses seen in the Mthal cells appear to be due to the inputs originating from these two different groups of PCs, although other factors such as the variation in the synaptic integration times may be contributing to the shape of the response.

Intuitively, if a cell receives projections predominantly from just one PC category, its spiking activity should exhibit a unimodal response, manifested as a single peak in the histogram. In contrast, when a cell receives projections from both PC categories, its spiking activity should display a bimodal distribution with two distinct peaks. However, in some of these cases, especially when the level of entrainment is low and asymmetric, the resulting activity may still appear unimodal and be classified as such by our analysis. For instance, Suppl. Fig. 4 illustrates this potential scenario with simulated spike histograms. This example features two histograms with a 180° phase shift (left and middle columns). The first histogram contains twice as many samples as the second, simulating an asymmetric response. The combined histogram has the appearance of a bimodal distribution (top right, PLV2 > PLV1) when the individual histograms are narrower and have a high level of entrainment (PLV=0.8). The combined histogram for the distributions with lower PLVs (bottom right, 0.5 each), however, presents a distribution that resembles more of a unimodal distribution (PLV1 > PLV2). These simulated cases demonstrate that a higher PLV increases the likelihood of the response being detected as a bimodal response as the distribution of each component becomes narrower and they overlap less. While this mechanism can explain the changes in modality with frequency, other dynamics within the CN and the thalamus may also contribute to the overall response.

Therefore, for the Mthal cells to switch from bimodal to unimodal distribution with varying frequencies, there are two prerequisites: First, the response must be bimodal at the origin, i.e. the thalamic cell (through the CN) receives inputs from both upward and downward PCs. Otherwise, purely unimodal inputs would result in a unimodal response across all frequencies. Second, the relative contributions from the two PC populations must be asymmetric since a perfectly symmetric bimodal input would produce bimodal Mthal responses in all frequencies as predicted by simulations (Suppl. Fig. 4).

These implications highlight two key points. First, the unimodal responses that we observe in the spiking activity of the thalamic cells may not imply that the PC inputs are predominantly upward or downward modulated at the origin. It is possible for a response driven by a mixed population of upward and downward PCs to appear unimodal at the CN or the thalamic level. This suggests that responses driven exclusively by either upward or downward PCs may be less common than suggested by the observed patterns in the Mthal cells. Second, when the response is a mixture of upward and downward PCs, the modulation may appear to be much lower at the Mthal cells than it is at the PC level.

We have to disclaim that our classification of responses as bimodal or unimodal is based solely on the appearance of the histograms and does not reveal the underlying population composition of upward and downward PCs. The observed shift in modality across frequencies reflects an increased likelihood of detecting dual peaks in the spike histogram at mid-range frequencies, probably driven by stronger entrainment, rather than indicating an actual shift in the balance of upward versus downward PC projections.

### C. Modulated and Unmodulated Thalamic Cells.

The staining with DiD’ in a few animals (example shown in Fig. 1) confirmed that the recorded thalamic cells were located within the VL/VM region. However, histological verification was performed only in a subset of animals, and thus the localization of all recorded units should be considered approximate. Nonetheless, there is supporting evidence from the literature that the cerebellar outputs primarily project to the VL. As shown by optogenetic stimulation in the rat, there is a higher density of CN axons terminating in the VL than VM [31]. Thalamocortical neurons in the VL with cerebellar afferents had higher tonic firing rates than those with basal ganglia afferents in *in vitro* slices from mice [32]. This agrees with our finding that the cells modulated by cerebellar stimulation had higher average firing rates than unmodulated cells (see bottom right, Suppl. Fig. 3). However, we are not aware of any reports on electrophysiological classification of the cells in the VL in anesthetized animals for direct comparison. The regular firing cells with short action potential duration may be a distinguishing feature of the cells in the VL/VM area with cerebellar afferents.

### D. MC Stimulation.

Interestingly, the bimodal responses commonly observed with cerebellar stimulation were very rare with MC stimulation (13 out of 164 trials). The rodent cerebellum, unlike its motor cortex, has gyrifications, which makes it possible for the cells to have different orientations inside the applied E-field. The orientation of the somatodendritic axis in the E-field determines the polarity and the strength of the induced transmembrane voltage [33]. The pyramidal cells in the output layer of the MC, however, may be experiencing the E-field in a much more uniform fashion than the PCs of the cerebellum and therefore give rise to primarily unimodal responses in the thalamic cells of their projection.

More than one peak in the PLV plot raises the possibility that entrainment of Mthal is occurring via multiple pathways having different frequency characteristics. Indeed, there exist multi-synaptic excitatory and inhibitory pathways from the MC [34], which can produce interfering inputs at the Mthal and maximize modulation at multiple frequencies where they add constructively. Another possibility is that one of the peaks reflects antidromic stimulation of thalamic axons that project to the MC. However, this is an unlikely scenario since suprathreshold stimulation would have very small variation in the arrival times of the action potentials invading the soma and the spike histogram would have an almost perfect entrainment, manifested as a sharp peak in the histogram.

Co-stimulation of the cerebellum along with motor and supplementary motor areas has been investigated for its potential to improve motor performance [15], [35], [36], [37]. In this paper, we looked at these potential modulatory inputs to the Mthal separately. Electrophysiological interference patterns between the inputs to the Mthal cells with tACS of the cerebellar and motor cortices (at 100 Hz and 110 Hz respectively) was investigated in a separate publication [38]. The insight gained from the electrophysiological data at single cell level can help determine the optimum frequencies and phase offsets to use for maximum clinical benefits with co-stimulation.

### E. PLV as a Measure of Entrainment.

The MPV and its magnitude (PLV) was used here to measure the level of spike entrainment. PLV was used in some cases to quantify entrainment at frequencies other than the stimulation frequency [39]. The PLV1 and PLV2 that we defined are analogous to the frequency components at the first and second harmonics of the stimulation frequency. The PLV metric, however, is upper-bounded and it reaches the maximum value of 1.0 when all the spikes fire at a single phase of the stimulation cycle. Thus, PLV quantifies how compact the spike timings accumulate around a specific phase, rather than looking for a sinusoidal appearance in the signal as in frequency domain transformations that use a set of sinusoidal base functions (e.g. Fourier Transforms). A histogram that peaks at both halves of the AC stimulation cycle will yield a high PLV2 (second harmonic) and a histogram with a single peak in the cycle will yield a high PLV1. Note that PLV2 is measured in half the time window length of PLV1. Thus, for PLV2 to have the same value as PLV1, the peaks in the histogram should be twice more compact or peaky. It should be kept in mind while reviewing our results that the PLV1 and PLV2 measure the sharpness of the histogram peaks in two different time windows.

## Supplementary Material

supp1-3644746
