# Supplementary material for: tACS of the Cerebellum and the Motor Cortex Entrains the Spiking Activity of the Cells in Motor Thalamus in a Frequency Dependent Manner
Source: IEEE Trans Neural Syst Rehabil Eng. Author manuscript; Available in PMC 2026 Feb 1. (PMC12861036; doi:10.1109/TNSRE.2025.3644746)
Supplement: supp1-3644746 [file NIHMS2135938-supplement-supp1-3644746.docx]

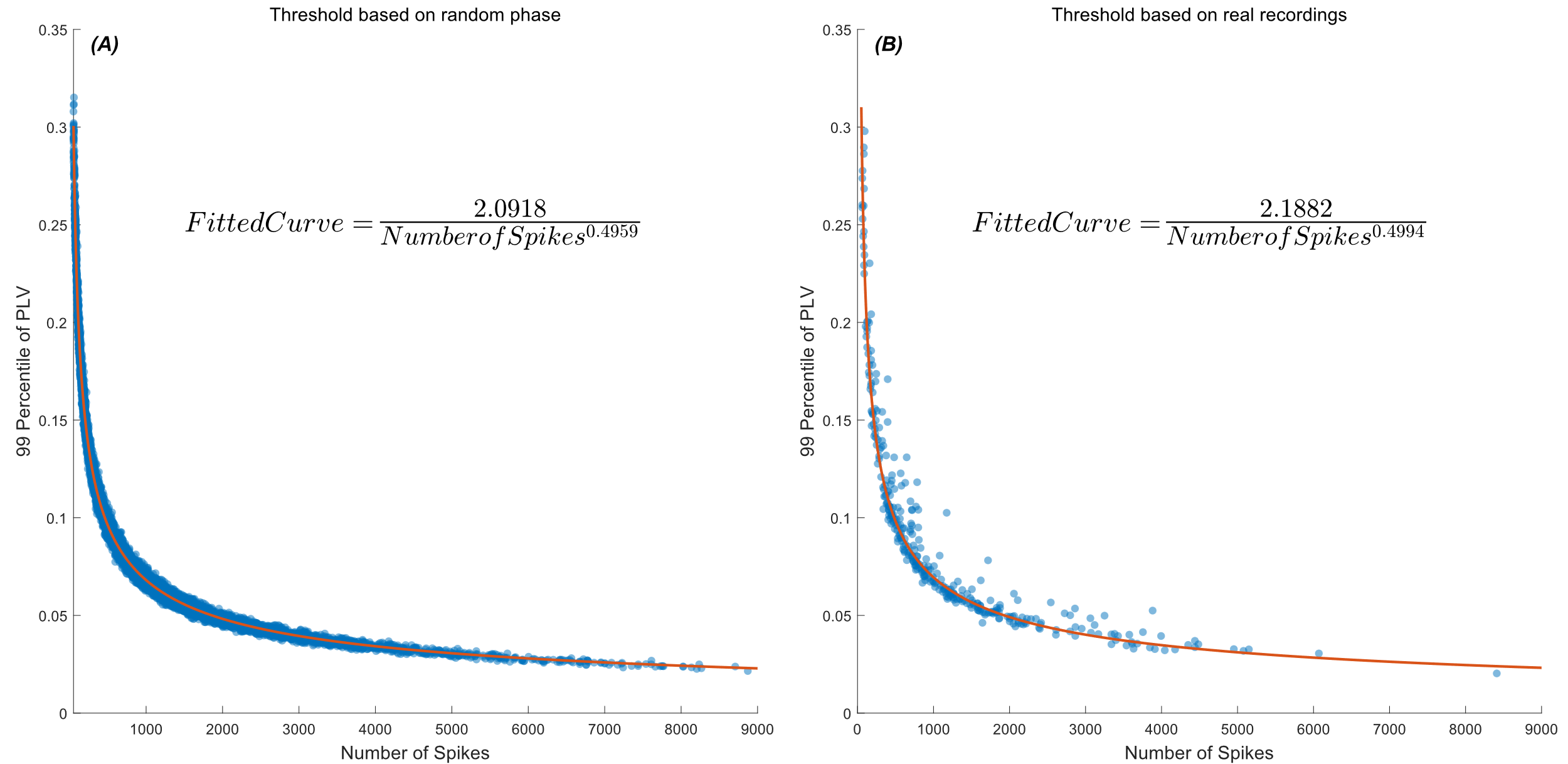


Suppl. Fig. 1. *PLV_Threshold_* formulations by curve-fitting to simulated and real neural data. ***A:*** 99 percentile of PLV distributions (1000 data points) for different Number of Spikes from simulated random spike times with uniform distribution. ***B:*** 99 percentile of the PLV distributions calculated from the spike times of 346 thalamic cells with artificially randomized phases. The equations that fit the simulated and real data are very similar.


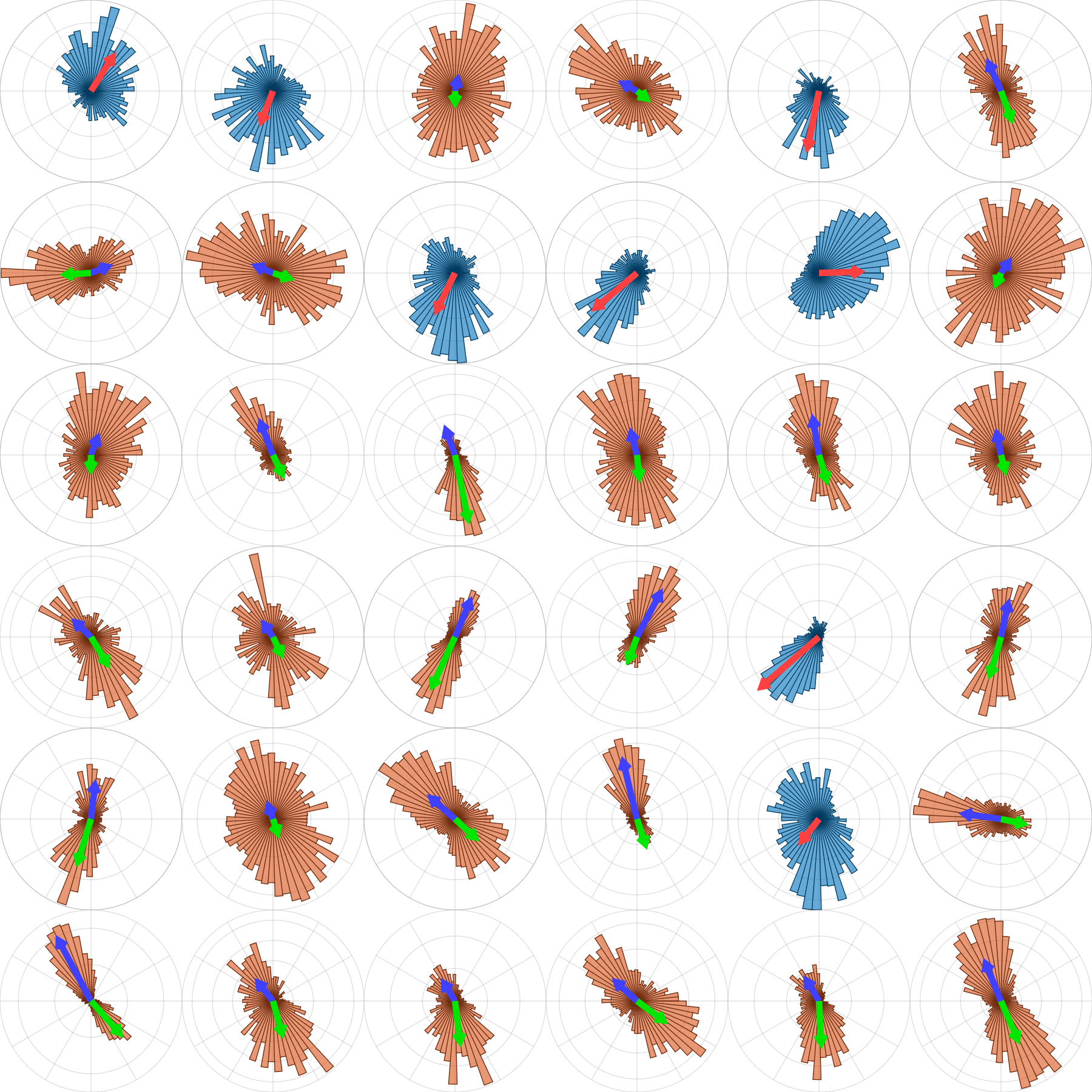


**Suppl. Fig. 2.** Histograms in the polar plane for 100 Hz AC stimulation in 36 (6x6 panels) thalamic cells. Histograms are shown in blue for unimodal responses and red for bimodal responses. The Mean Phase Vectors are drawn as arrows, where the arrow length represents the PLV and the arrow angle is the PPh. For bimodal responses, two vectors (blue and green) are plotted to represent each of the two out-of-phase groups in the histogram separately. In this case the vectors amplitude represents the PLV2 scaled by the number of spikes in each group to illustrate the degree of asymmetry between the two groups and their angle represents the PPh for each group.


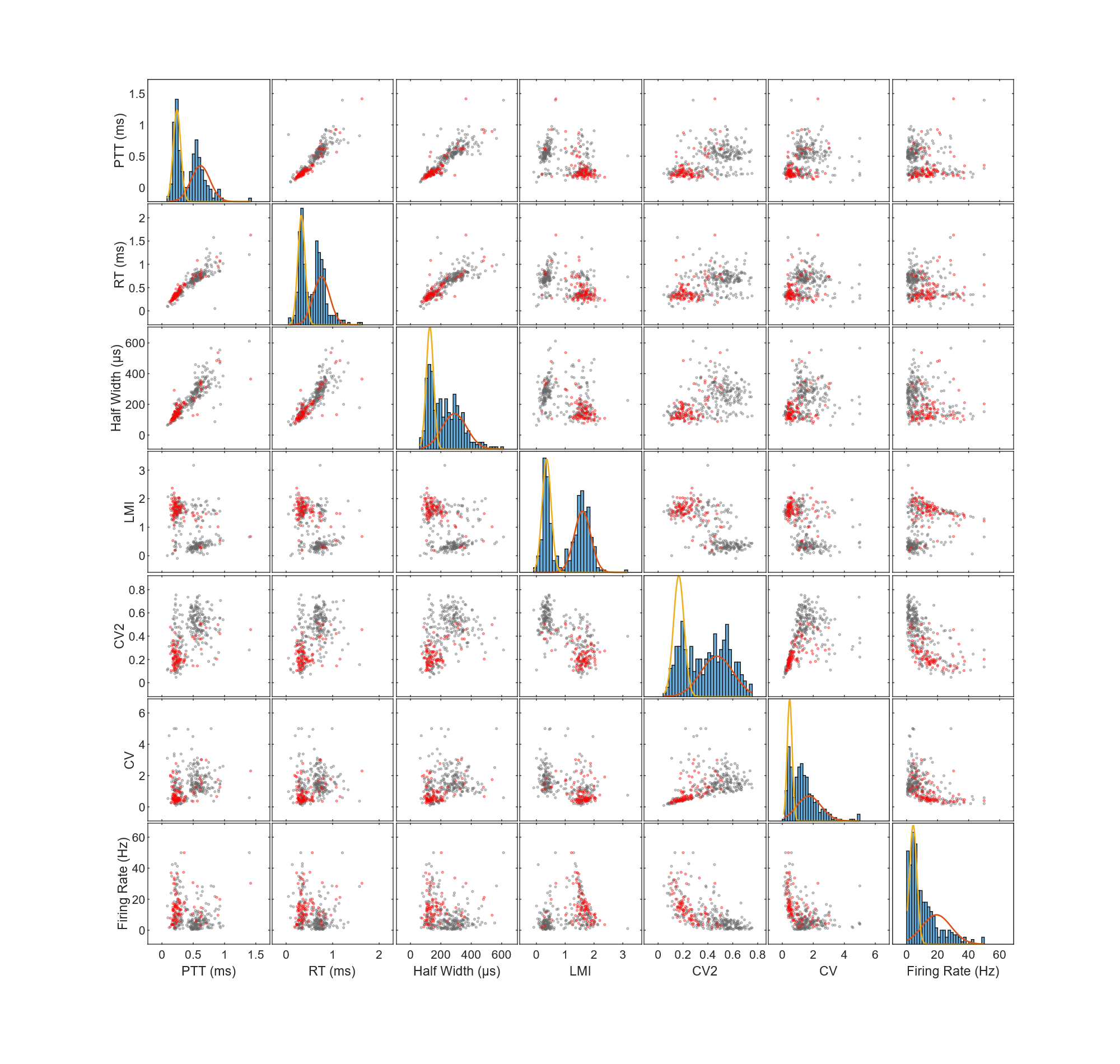


**Suppl. Fig. 3**. Distribution of different parameters against each other in pairs, and their individual histogram (diagonal boxes). Histogram for each parameter is fitted with two normal distributions.

To divide the distribution of each parameter into two clusters, first a kernel smoothing function (*ksdensity*) was applied using a bandwidth set to 2% of the data range. The trough (local minimum) in the resulting density estimate was then used as the division point to separate the data into two clusters. After this division, a Gaussian distribution was fitted to each subset individually.


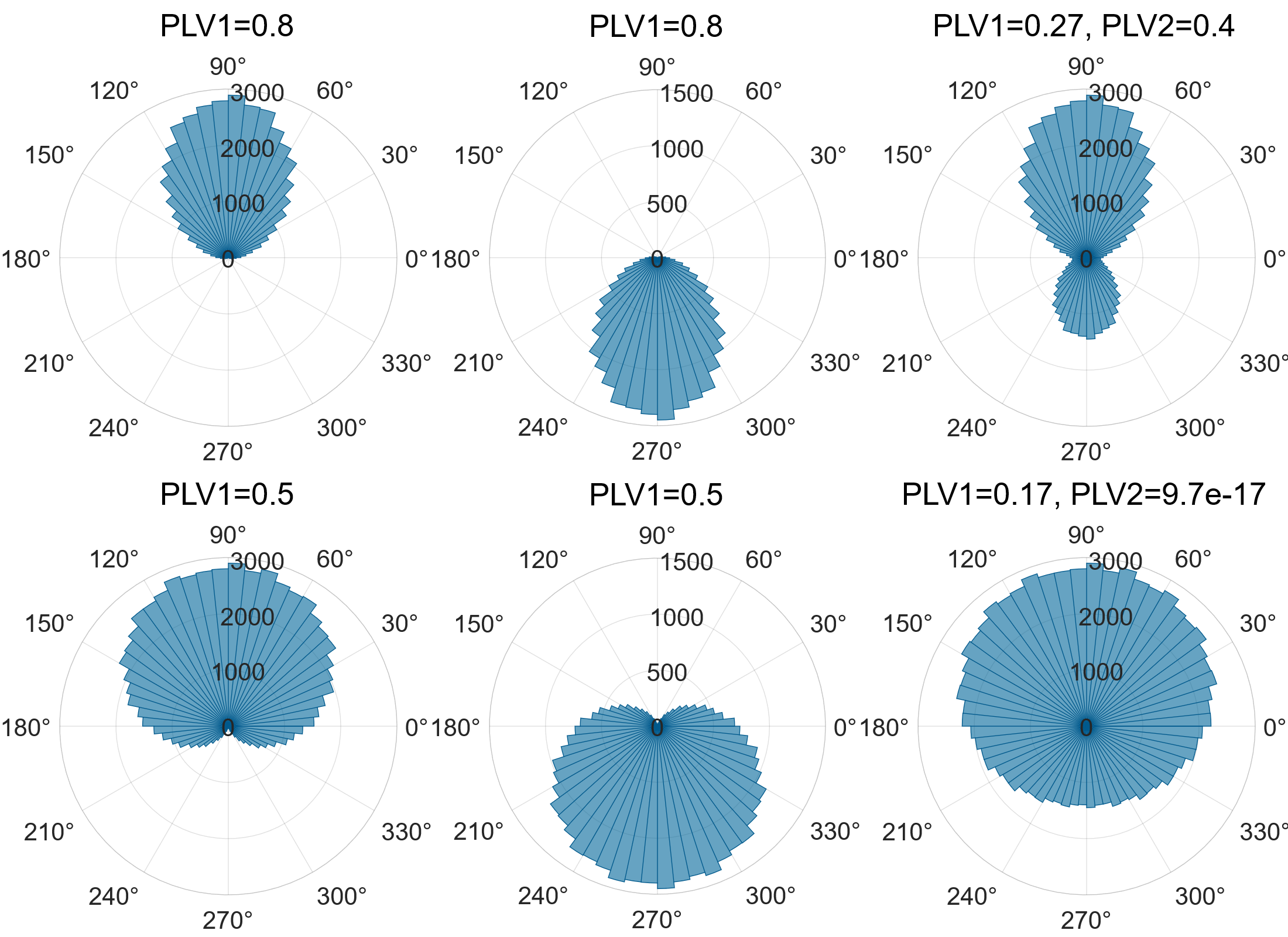


**Suppl. Fig. 4.** Theoretical comparison of histograms of bimodal distributions that can be constructed as combinations of unimodal histograms with high (top row) and moderate PLV (bottom row) and asymmetric number of spikes (i.e. first column has two times higher counts than the second column). The polar histograms in the rightmost column show the combined plots of the two unimodal plots to their left. The combined plot for two unimodal PLVs at moderate level (bottom right) does not have the appearance of a bimodal distribution as it does with that of high PLVs (top right) and yields a higher PLV1 than PLV2.
